# Characteristics and Prescribing Patterns of Clinicians Waivered to Prescribe Buprenorphine for Opioid Use Disorder Before and After Release of New Practice Guidelines

**DOI:** 10.1001/jamahealthforum.2023.1982

**Published:** 2023-07-21

**Authors:** Christopher M. Jones, Yngvild Olsen, Mir M. Ali, Tisamarie B. Sherry, Jana Mcaninch, Timothy Creedon, Patti Juliana, Laura Jacobus-Kantor, Robert Baillieu, Mamadou Misbaou Diallo, Anita Thomas, Neeraj Gandotra, Marta Sokolowska, Shari Ling, Wilson Compton

**Affiliations:** 1 Centers for Disease Control and Prevention, Atlanta, Georgia; 2 Substance Abuse and Mental Health Services Administration, Rockville, Maryland; 3 Office of the Assistant Secretary for Planning and Evaluation, US Department of Health and Human Services, Washington, DC; 4 US Food and Drug Administration, Silver Spring, Maryland; 5 Centers for Medicare & Medicaid Services, Baltimore, Maryland; 6 National Institutes of Health, Bethesda, Maryland

## Abstract

**Importance:**

In April 2021, the US Department of Health and Human Services (HHS) released practice guidelines exempting educational requirements to obtain a Drug Addiction Treatment Act (DATA) waiver to treat up to 30 patients with opioid use disorder with buprenorphine.

**Objective:**

To compare demographic and practice characteristics of clinicians who received traditional DATA waivers before and after release of the education-exempted HHS practice guidelines and those who were approved under the guidelines.

**Design, Setting, and Participants:**

This survey study was conducted electronically from February 1 to March 1, 2022. Eligible survey recipients were US clinicians who obtained an initial DATA waiver between April 2020 and November 2021.

**Exposure:**

DATA waiver approval pathway.

**Main Outcome and Measures:**

The outcomes were clinician demographic and practice characteristics, buprenorphine prescribing barriers, and strategies to treat patients with opioid use disorder, measured using χ^2^ tests and *z* tests to assess for differences among the waivered groups.

**Results:**

Of 23 218 eligible clinicians, 4519 (19.5%) responded to the survey. This analysis was limited to 2736 respondents with a 30-patient limit at the time of survey administration who identified their DATA waiver approval pathway. Among these respondents, 1365 (49.9%; female, 831 [61.9%]; male, 512 [38.1%]) received their DATA waiver prior to the education-exempted practice guidelines (prior DATA waiver), 550 (20.1%; female, 343 [63.4%]; male, 198 [36.6%]) received their waiver after guidelines were released but met education requirements (concurrent DATA waiver), and 821 (30.0%; female, 396 [49.2%]; male, 409 [50.8%]) received the waiver under the education-exempted guidelines (practice guidelines). Among practice guidelines clinicians, 500 (60.9%) reported that traditional DATA waiver educational requirements were a reason for not previously obtaining a waiver. Demographic and practice characteristics differed by waiver approval type. Across all groups, a large minority had not prescribed buprenorphine since obtaining a waiver (prior DATA waiver, 483 [35.7%]; concurrent DATA waiver, 226 [41.2%]; practice guidelines, 359 [44.3%]; *P* < .001). Clinicians who prescribed buprenorphine in the past 6 months reported treating few patients in an average month: 27 practice guidelines clinicians (6.0%) prescribed to 0 patients and 338 (75.1%) to 1 to 4 patients compared with 16 (2.2%) and 435 (59.9%) for prior and 11 (3.6%) and 166 (55.0%) for concurrent DATA waiver clinicians, respectively (*P* < .001). Across waiver types, clinicians reported multiple challenges to buprenorphine prescribing.

**Conclusions and Relevance:**

In this survey of DATA-waivered clinicians, clinician- and systems-level challenges that limit buprenorphine prescribing were observed, even among clinicians approved under the education-exempted guidelines pathway. The findings suggest that as implementation of legislation removing the DATA waiver begins, addressing these barriers could be essential to increasing buprenorphine access.

## Key Points

**Question:**

In what way do the characteristics of clinicians who received Drug Addiction Treatment Act (DATA) waivers under the 2021 education-exempted US Department of Health and Human Services buprenorphine practice guidelines differ from those who obtained their DATA waiver through traditional waiver approval pathways?

**Findings:**

In this survey study of 2736 clinicians, those waivered under the education-exempted guidelines were less likely than traditionally waivered clinicians to prescribe buprenorphine and more likely to practice in emergency or urgent care settings. The survey identified multiple barriers to buprenorphine prescribing across waiver approval types.

**Meaning:**

The findings suggest that multipronged efforts may be needed to focus on addressing clinician-, patient-, and systems-level barriers to prescribing buprenorphine.

## Teaser

This survey study compares characteristics and buprenorphine prescribing patterns for opioid use disorder (OUD) among clinicians before and after the release of new practice guidelines.

## Introduction

Buprenorphine, a highly effective treatment for opioid use disorder (OUD), is the most accessible form of medication for OUD (MOUD) in the US^1,2^ but remains substantially underused.^2,3^ Historically, only clinicians who obtained a Drug Addiction Treatment Act (DATA) waiver could prescribe buprenorphine for OUD treatment.^2,4^ Most eligible clinicians did not obtain such a waiver, and research identified educational training requirements as 1 reason for this lack of uptake.^5,6,7^ With passage of the Consolidated Appropriations Act of 2023, the US Congress legislatively removed the DATA waiver to prescribe buprenorphine for OUD.^8^ Although this change has the potential to substantially increase access to buprenorphine treatment, prior research has shown that there are multiple clinician-, patient-, and systems-level barriers to care independent of the DATA waiver that may continue to limit buprenorphine prescribing.^3,5,9,10^

Assessing the outcomes of regulatory action by the US Department of Health and Human Services (HHS) in April 2021 aimed at removing the educational training requirement prior to obtaining a DATA waiver may provide important insight into how the recent legislative change of fully removing the DATA waiver might change clinical practice with respect to buprenorphine prescribing and what barriers to prescribing remain even after removal of the educational requirement. On April 27, 2021, the HHS released its *Practice Guidelines for the Administration of Buprenorphine for Treating Opioid Use Disorder*,^4^ which provides an additional pathway to obtain a DATA waiver by exempting eligible clinicians from the federal education requirements needed to obtain a DATA waiver to treat up to 30 concurrent patients with buprenorphine. Clinicians, including those interested in treating more than 30 patients concurrently, could still obtain a traditional DATA waiver after release of the practice guidelines.

A recent study found that the HHS practice guidelines were associated with an acceleration in the growth of clinicians obtaining a 30-patient-limit DATA waiver,^11^ consistent with other research that showed increases in numbers of DATA-waivered clinicians after release of the practice guidelines.^12,13^ The analysis also found that this increase in waivered clinicians did not translate into an acceleration in patients filling buprenorphine prescriptions above historical trends.^11^ Importantly, these studies^11,12,13^ could not examine how clinicians seeking a waiver under the education-exempted practice guidelines were similar to or different from those seeking a traditional waiver before the release of the practice guidelines or those obtaining a traditional waiver after the release of the practice guidelines, a group completing educational requirements despite no longer being required. Furthermore, prior studies have not examined buprenorphine prescribing practices or barriers to prescribing MOUD and how these compare with traditionally waivered clinicians. To address this research gap, we conducted a survey to compare traditionally waivered clinicians before or concurrent with release of the education-exempted practice guidelines and those waivered under the practice guidelines in order to gain actionable insights as policy makers and the clinical community begin implementation of buprenorphine treatment without the need for the DATA waiver.

## Methods

### Study Sample and Data Collection

We conducted a cross-sectional survey study of 3 subpopulations of clinicians: (1) clinicians obtaining a traditional 30-patient-limit DATA waiver in the 12 months prior to the release of the HHS practice guidelines (April 2020 to April 2021); (2) clinicians receiving a traditional 30-patient-limit DATA waiver during the 6 months following the release of the guidelines (June 2021 to November 2021); and (3) clinicians who received a DATA waiver under the guidelines during the same 6-month period (June 2021 to November 2021). Data on clinicians with a DATA waiver were from the Drug Enforcement Administration’s controlled substances registration database and based on the date the clinician received their initial 30-patient waiver. Initial emails with an embedded survey link were sent to each eligible clinician using REDCap^14^ (Vanderbilt University) on February 1, 2022, with reminder emails 1.5 and 3 weeks later; the survey closed after 4 weeks on March 1, 2022. The Centers for Disease Control and Prevention determined this investigation to be public health surveillance and exempt from institutional review board review by regulation.^15^ The email invitation to participate in the survey indicated that by participating, the respondent provides informed consent. This study followed the American Association for Public Opinion Research (AAPOR) recommendations for conducting surveys, including method of survey delivery, survey design and testing, and reporting results.^16^

### Survey Design

The 45-question survey instrument (eTable 1 in Supplement 1) was based on prior surveys examining buprenorphine prescribing and barriers to prescribing^9,10^ and expert review. The survey included questions in 3 domains: (1) clinician demographic and practice characteristics, (2) buprenorphine prescribing practices and barriers to prescribing, and (3) challenges faced and strategies used to treat patients with OUD. Survey respondents self-reported data on demographic characteristics from provided categories, including gender identity (female; male; nonbinary/genderqueer/gender nonconforming/neither exclusively male or female; and other), race (American Indian or Alaska Native, Asian, Black or African American, Native Hawaiian or other Pacific Islander, and White), and ethnicity (Hispanic or Latino and not Hispanic or Latino). These variables were of interest to identify potential demographic disparities by clinician type and waiver approval type, an area that has been underexplored in previous research.

### Statistical Analysis

We report descriptive statistics for clinician demographic and practice characteristics overall and by the 3 independent groups based on DATA waiver type (traditional vs HHS practice guidelines) and timing of receipt of their waiver as follows: (1) traditional waiver before HHS practice guidelines (hereafter prior DATA waiver), (2) traditional waiver after HHS practice guidelines (hereafter concurrent DATA waiver), and (3) waiver under education-exempted HHS practice guidelines (hereafter practice guidelines). We calculated frequencies and percentages for questions related to buprenorphine prescribing practices, barriers to remote prescribing, challenges faced, and strategies used to treat patients with OUD among clinicians who reported prescribing buprenorphine in the past 6 months or since obtaining a waiver under the practice guidelines.

We conducted all analyses with Stata, version 15.1 (StataCorp LLC) statistical software using χ^2^ tests and *z*-tests to assess for statistically significant differences among groups based on waiver type. Two-tailed *P* < .05 was considered statistically significant.

## Results

### Demographic and Clinical Practice Characteristics

Of the 23 218 eligible clinicians receiving the survey, 4519 responded (19.5%), with 2736 (60.5%) providing sufficient information to be included in this specific analysis; some survey items were not answered by every clinician. The geographic distribution of eligible survey recipients and ultimate respondents closely matched (eTable 2 in Supplement 1). In addition, the percentage of respondents across the 3 waiver approval groups was similar to the percentage of eligible survey recipients in each group (prior DATA waiver, 14 847 [59.5%] vs 1926 [58.4%]; concurrent DATA waiver, 3244 [13.0%] vs 550 [16.7%]; practice guidelines, 6872 [27.5%] vs 821 [24.9%]).

This analysis is limited to the subset of 2736 respondents who indicated that they still had a 30-patient limit at the time of survey administration and identified their DATA waiver approval pathway (eFigure in Supplement 1). Among this group, 1365 (49.9%) were prior DATA waiver clinicians (female, 831 [61.9%]; male, 512 [38.1%]; American Indian or Alaska Native, 3 [0.5%]; Asian or Pacific Islander, 130 [9.9%]; Black, 151 [11.5%]; Hispanic, 91 [7.0%]; White, 896 [68.5%]; multiracial, 34 [2.6%]), 550 (20.1%) were concurrent DATA waiver clinicians (female, 343 [63.4%]; male, 198 [36.6%]; American Indian or Alaska Native, 3 [0.6%]; Asian or Pacific Islander, 53 [9.9%]; Black, 86 [16.1%]; Hispanic, 35 [6.6%]; White, 342 [64.0%]; multiracial, 15 [2.8%]), and 821 (30.0%) were practice guidelines clinicians (female, 396 [49.2%]; male, 409 [50.8%]; American Indian or Alaska Native, 2 [0.3%]; Asian or Pacific Islander, 98 [12.5%]; Black, 35 [4.5%]; Hispanic, 44 [5.6%]; White, 584 [74.3%]; multiracial, 23 [2.9%]) (Table). Multiple clinical practice characteristics differed between practice guidelines clinicians and both traditionally waivered groups. For example, a larger percentage of practice guidelines (vs prior and concurrent DATA waiver) clinicians were physicians (rather than physician assistants or nurse practitioners) (628 [76.8%] vs 768 [56.4%] and 264 [48.1%]), practiced in urban (416 [51.3%] vs 606 [44.9%] and 242 [44.6%]) or suburban (271 [33.4%] vs 404 [29.9%] and 151 [27.8%]) areas, practiced in emergency department (ED) or urgent care settings (326 [39.9%] vs 230 [17.0%] and 68 [12.5%]), reported not being listed on the Substance Abuse and Mental Health Services Administration’s (SAMHSA’s) buprenorphine locator (254 [31.3%] vs 345 [25.6%] and 136 [25.0%]), and reported never interacting with SAMHSA’s Providers Clinical Support System for Medication Assisted Treatment (615 [75.4%] vs 869 [63.9%] and 350 [64.1%]).

**Table.** Demographic and Practice Characteristics of Clinicians Responding to the Survey

Variable	No. (%)^a^			*P* value
	DATA waiver		Practice guidelines	
	Prior	Concurrent		
Total sample	1365 (49.9)	550 (20.1)	821 (30.0)	NA
Gender identity				
Female	831 (61.9)	343 (63.4)	396 (49.2)	<.001
Male	512 (38.1)	198 (36.6)	409 (50.8)	
Age, y				
≤34	272 (20.2)	114 (21.0)	131 (16.1)	.23
35-44	418 (31.0)	168 (31.0)	249 (30.6)	
45-54	318 (23.6)	137 (25.3)	217 (26.7)	
55-64	228 (16.9)	88 (16.2)	145 (17.8)	
≥65	113 (8.4)	35 (6.5)	71 (8.7)	
Race and ethnicity				
Hispanic	91 (7.0)	35 (6.6)	44 (5.6)	<.001
Non-Hispanic				
American Indian or Alaska Native	6 (0.5)	3 (0.6)	2 (0.3)	
Asian or Pacific Islander	130 (9.9)	53 (9.9)	98 (12.5)	
Black	151 (11.5)	86 (16.1)	35 (4.5)	
White	896 (68.5)	342 (64.0)	584 (74.3)	
Multiracial	34 (2.6)	15 (2.8)	23 (2.9)	
Census region				
Northeast	263 (19.4)	105 (19.2)	151 (18.4)	<.001
Midwest	332 (24.5)	140 (25.6)	149 (18.2)	
South	333 (24.5)	149 (27.2)	192 (23.4)	
West	429 (31.6)	153 (28.0)	327 (39.9)	
Primary practice location				
Urban	606 (44.9)	242 (44.6)	416 (51.3)	<.001
Suburban	404 (29.9)	151 (27.8)	271 (33.4)	
Rural	340 (25.2)	150 (27.6)	124 (15.3)	
Clinician type				
Physician (MD/DO)	768 (56.4)	264 (48.1)	628 (76.8)	<.001
NP or other nurse-eligible practitioner	499 (36.6)	246 (44.8)	143 (17.5)	
PA	95 (7.0)	39 (7.1)	47 (5.7)	
Primary practice specialty				
Primary care, internal medicine, or family medicine	697 (51.3)	295 (53.8)	321 (39.2)	<.001
Pediatrics	14 (1.0)	4 (0.7)	9 (1.1)	
Obstetrics and gynecology	48 (3.5)	10 (1.8)	15 (1.8)	
Emergency medicine	228 (16.8)	57 (10.4)	329 (40.1)	
Psychiatry or addiction medicine	263 (19.4)	140 (25.6)	100 (12.2)	
Pain medicine, anesthesiology, physical medicine, or rehabilitation	34 (2.5)	18 (3.3)	13 (1.6)	
Palliative care and hospice care	19 (1.4)	7 (1.3)	10 (1.2)	
Other	56 (4.1)	17 (3.1)	23 (2.8)	
Addiction medicine credentials				
No	1343 (98.4)	539 (98.0)	808 (98.4)	.81
Yes	22 (1.6)	11 (2.0)	13 (1.6)	
Years in practice since completing residency (MD/DO) or qualifying program (PA, NP, or other nurse-eligible practitioner)				
<5	562 (41.4)	266 (48.6)	207 (25.3)	<.001
5-10	268 (19.7)	96 (17.6)	164 (20.1)	
11-15	149 (11.0)	53 (9.7)	115 (14.1)	
16-19	86 (6.3)	32 (5.9)	82 (10.0)	
≥20	294 (21.6)	100 (18.3)	250 (30.6)	
Primary practice setting				
Office-based solo practice	106 (7.8)	46 (8.4)	46 (5.6)	<.001
Office-based group practice	386 (28.5)	167 (30.6)	158 (19.3)	
Opioid treatment program or specialty substance use facility	29 (2.1)	25 (4.6)	9 (1.1)	
Community clinic (FQHC, RHC, CCBHC)	232 (17.1)	97 (17.8)	72 (8.8)	
ED or urgent care	230 (17.0)	68 (12.5)	326 (39.9)	
Criminal justice setting (including BOP)	32 (2.4)	14 (2.6)	14 (1.7)	
Inpatient or hospital setting (not federal government)	160 (11.8)	60 (11.0)	123 (15.0)	
Federal government (VA, DOD, or IHS)	76 (5.6)	31 (5.7)	29 (3.6)	
Other (specify)	103 (7.6)	37 (6.8)	41 (2.0)	
Insurance type accepted				
Medicaid	1128 (82.6)	443 (80.6)	701 (85.4)	.06
Medicare	1134 (83.1)	453 (82.4)	700 (85.3)	.28
Workers’ compensation	529 (38.8)	189 (34.4)	417 (50.8)	<.001
Private, commercial, other insurance	1167 (85.5)	486 (88.4)	720 (87.7)	.15
Do not accept any type of insurance in my practice	81 (5.9)	27 (4.9)	24 (2.9)	.006
Listed on SAMHSA buprenorphine locator				
No	345 (25.6)	136 (25.0)	254 (31.3)	<.001
Yes	444 (32.9)	212 (38.9)	151 (18.6)	
Do not know	560 (41.5)	197 (36.2)	407 (50.1)	
Ever interacted with PCSS-MAT				
No	869 (63.9)	350 (64.1)	615 (75.4)	<.001
Yes	288 (21.2)	107 (19.6)	76 (9.3)	
Do not know	203 (14.9)	89 (16.3)	125 (15.3)	
Sources of education about buprenorphine for OUD treatment and/or OUD diagnosis and treatment				
Medical or professional school teaching	460 (33.7)	175 (31.8)	274 (33.4)	.73
Postgraduate residency training	260 (19.1)	91 (16.6)	184 (22.4)	.02
Fellowship training	60 (4.4)	20 (3.6)	34 (4.1)	.75
Scientific conferences or continuing education	751 (55.0)	237 (43.1)	414 (50.4)	<.001
Self-directed reading of published literature or guidelines	845 (61.9)	321 (58.4)	571 (69.6)	<.001
Promotional materials or detailing from drug companies (not FDA REMS–related materials)	107 (7.8)	39 (7.1)	41 (5.0)	.04
FDA REMS materials related to buprenorphine products for OUD (eg, REMS instruction letter to prescribers, medication guide, appropriate use checklist)	371 (27.2)	136 (24.7)	171 (20.8)	.004
Colleagues (not part of formal training or continuing education)	591 (43.3)	219 (39.8)	466 (56.8)	<.001
Other	72 (5.3)	29 (5.3)	33 (4.0)	.38
None of the above	55 (4.0)	27 (4.9)	29 (3.5)	.45
Prescribed buprenorphine since obtaining waiver				
No	483 (35.7)	226 (41.2)	359 (44.3)	<.001
Yes	869 (64.3)	322 (58.8)	451 (55.7)	
Prescribed buprenorphine in past 6 mo				
No	619 (45.9)	243 (44.4)	359 (44.3)	.72
Yes	729 (54.1)	304 (55.6)	451 (55.7)	
Remote buprenorphine prescribing to new patient^b^				
No	625 (85.7)	265 (87.2)	422 (93.6)	<.001
Yes	104 (14.3)	39 (12.8)	29 (6.4)	

Abbreviations: BOP, Federal Bureau of Prisons; CCBHC, certified community behavioral health clinic; DATA, Drug Addiction Treatment Act; DO, doctor of osteopathic medicine; DOD, US Department of Defense; ED, emergency department; FDA REMS, US Food and Drug Administration Risk Evaluation and Mitigation Strategy; FQHC, federally qualified health center; IHS, Indian Health Service; MD, doctor of medicine; NA, not applicable; NP, nurse practitioner; OUD, opioid use disorder; PA, physician assistant; PCSS-MAT, Providers Clinical Support System for Medication Assisted Treatment; RHC, rural health clinic; SAMHSA, Substance Abuse and Mental Health Services Administration; VA, US Department of Veterans Affairs.

^a^ Respondents had the ability to skip specific demographic and clinical practice characteristic questions. Thus, responses for an individual characteristic may not sum to the total number of respondents for each waiver approval type category.

^b^ Among clinicians who reported prescribing in the past 6 months for those obtaining a traditional DATA waiver or since obtaining a waiver under the practice guidelines.

More than one-third of clinicians in each group reported not prescribing buprenorphine since obtaining their DATA waiver (prior DATA waiver, 483 [35.7%]; concurrent DATA waiver, 226 [41.2%]; practice guidelines, 359 [44.3%]; *P* < .001) (Table). The percentage of clinicians who reported prescribing buprenorphine in the past 6 months was similar (prior DATA waiver, 729 [54.1%]; concurrent DATA waiver, 304 [55.6%]; practice guidelines, 451 [55.7%]; *P* = .72). Fewer practice guidelines clinicians (29 [6.4%]) reported prescribing buprenorphine remotely to a new patient in the past 6 months vs prior DATA waiver (104 [14.3%]) and concurrent DATA waiver (39 [12.8%]) clinicians (*P* < .001).

### Reasons for Not Obtaining a Traditional DATA Waiver

**Figure 1.**
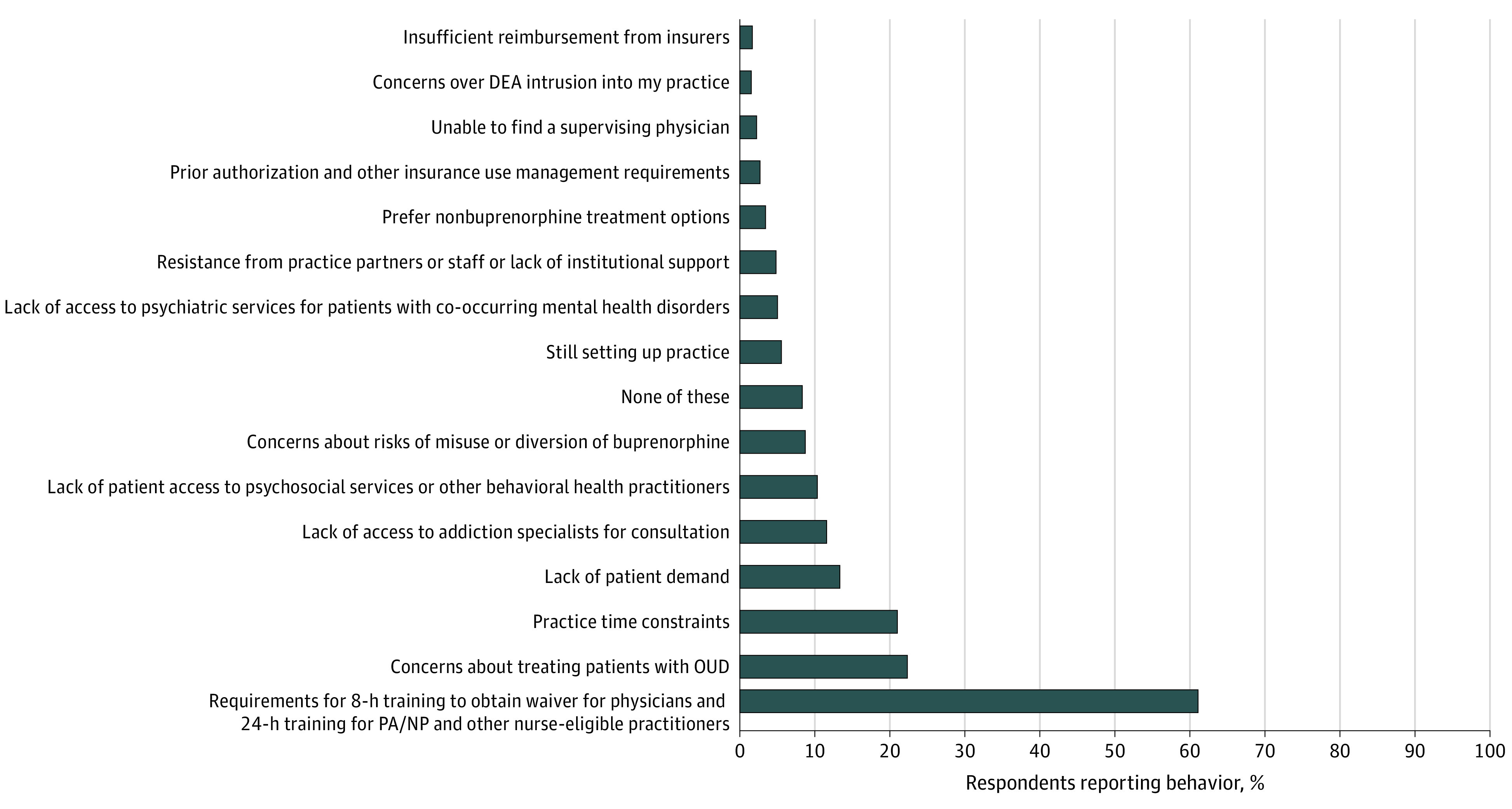
Reasons for Not Obtaining a Traditional Drug Addiction Treatment Act Waiver Among Clinicians Approved Under the Education-Exempted Practice Guidelines DEA indicates Drug Enforcement Administration; NP, nurse practitioner; OUD, opioid use disorder; PA, physician assistant.

Among practice guidelines clinicians, 500 (60.9%) indicated that requirements for educational training for the traditional DATA waiver was a reason for not previously obtaining a traditional DATA waiver (Figure 1). Other commonly cited reasons for not previously obtaining a waiver by this group included concerns about treating patients with OUD (184 [22.4%]), practice time constraints (172 [21.0%]), lack of patient demand (105 [12.8%]), lack of access to addiction specialists for consultation (97 [11.8%]), and lack of patient access to psychological services or other behavioral health practitioners (86 [10.5%]).

### Reasons for Not Prescribing Buprenorphine Since Obtaining DATA Waiver

**Figure 2.**
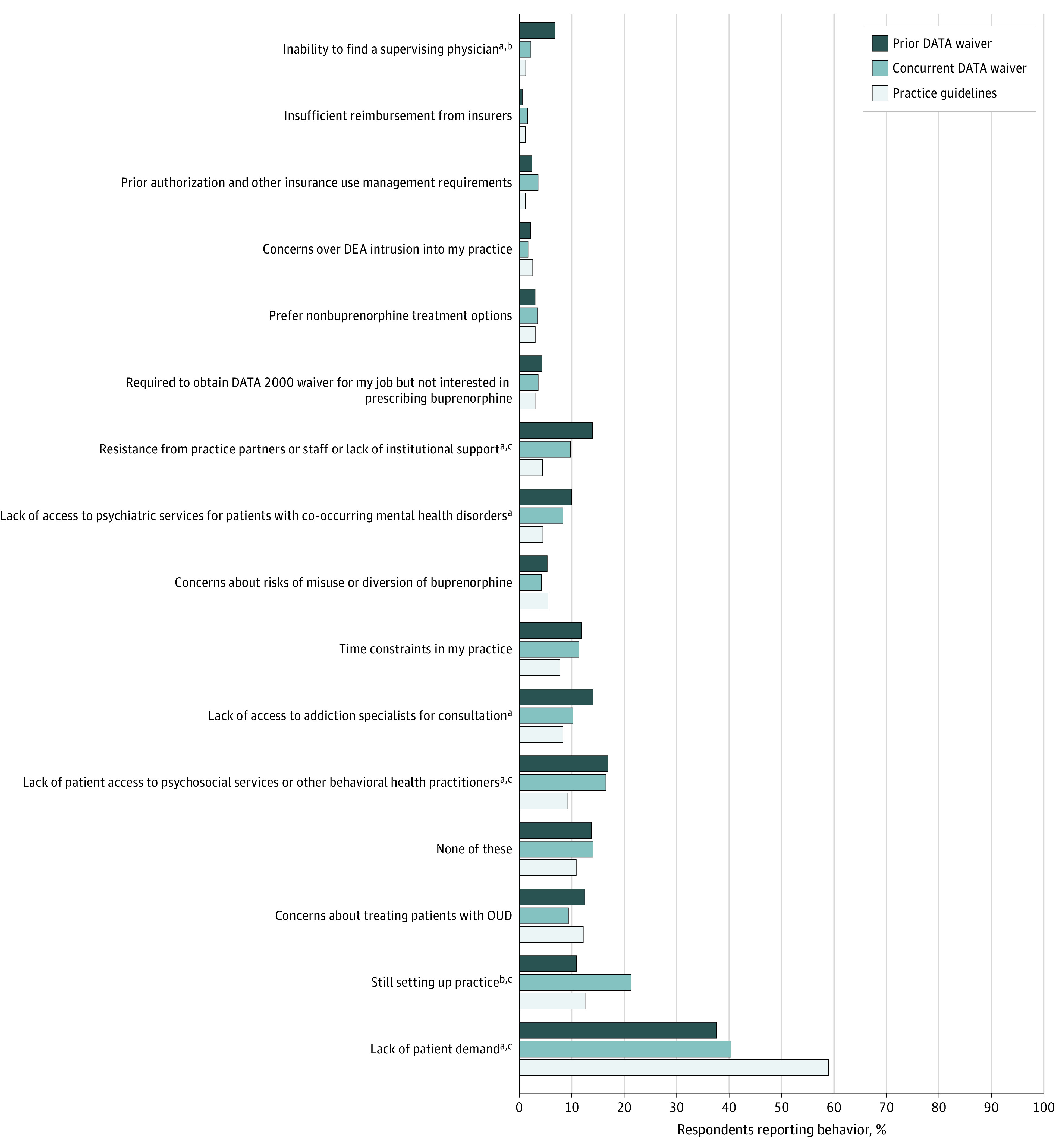
Reasons for Not Prescribing Buprenorphine for Opioid Use Disorder Since Obtaining a Drug Addiction Treatment Act (DATA) Waiver DEA indicates Drug Enforcement Administration; OUD, opioid use disorder. ^a^Significant difference between practice guidelines and prior DATA waiver. ^b^Significant difference between prior DATA waiver and concurrent DATA waiver. ^c^Significant difference between practice guidelines and concurrent DATA waiver.

Among respondents who had not prescribed buprenorphine since obtaining their DATA waiver, reasons for not prescribing varied (Figure 2). Lack of patient demand was the most common reason cited across groups (prior DATA waiver, 183 [37.9%]; concurrent DATA waiver, 91 [40.3%]; practice guidelines, 213 [59.3%]; *P* < .001). The following reasons also significantly differed between practice guidelines and prior DATA waiver clinicians: lack of patient access to psychosocial services or other behavioral health practitioners (34 [9.5%] vs 82 [17.0%]; *P* = .001); lack of access to addiction specialists for consultation (30 [8.4%] vs 68 [14.1%]; *P* = .008); lack of access to psychiatric services for patients with co-occurring mental health disorders (16 [4.5%] vs 49 [10.1%]; *P* = .001); resistance from practice partners or staff or lack of institutional support (16 [4.5%] vs 68 [14.1%]; *P* < .001); and inability to find a supervising physician (3 [0.8%] vs 33 [6.8%]; *P* < .001). Significant differences between practice guidelines and concurrent DATA waiver clinicians included still setting up practice (46 [12.8%] vs 49 [21.7%]; *P* = .007); lack of patient access to psychosocial services or other behavioral health practitioners (34 [9.5%] vs 38 [17.0%]; *P* = .01); and resistance from practice partners or staff or lack of institutional support (16 [4.5%] vs 22 [9.7%]; *P* = .02). Still setting up practice (49 [21.7%] vs 55 [11.4%]; *P* < .001) was more common and inability to find a supervising physician (5 [2.2%] vs 33 [6.8%]; *P* = .02) was less common among concurrent DATA waiver clinicians compared with prior DATA waiver clinicians.

### Number of Patients Prescribed Buprenorphine

**Figure 3.**
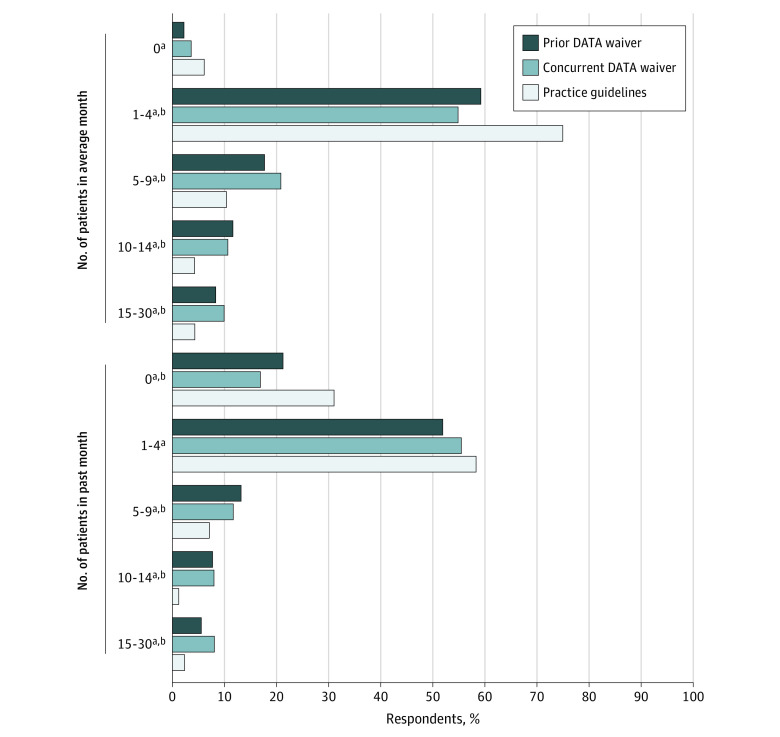
Number of Patients Prescribed Buprenorphine in an Average Month Over the Past 6 Months and in the Past Month ^a^Significant difference between practice guidelines and prior Drug Addiction Treatment Act (DATA) waiver. ^b^Significant difference between practice guidelines and concurrent DATA waiver.

Most clinicians who had prescribed buprenorphine in the past 6 months prescribed to 1 to 4 patients in an average month and in the past month, regardless of waiver approval type (Figure 3). In general, a larger percentage of practice guidelines clinicians compared with prior and concurrent DATA waiver clinicians reported prescribing to either 0 patients (27 [6.0%] vs 16 [2.2%] and 11 [3.6%]) or 1 to 4 patients (338 [75.1%] vs 435 [59.9%] and 166 [55.0%]) in an average month (*P* < .001). There were no significant differences between prior and concurrent DATA waiver groups with respect to number of patients prescribed buprenorphine in the past month or in an average month.

### Strategies to Monitor and Engage Patients Prescribed Buprenorphine

**Figure 4.**
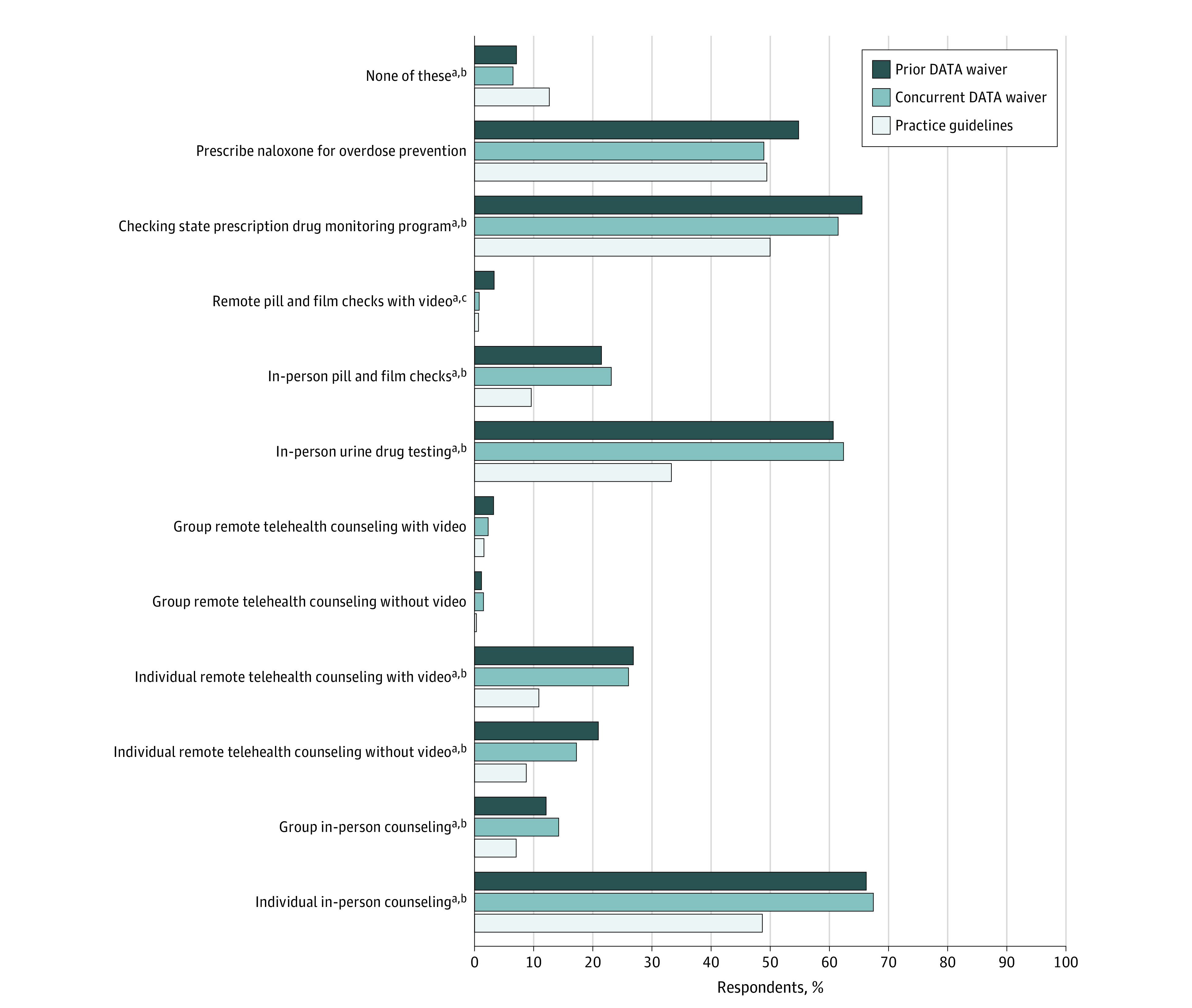
Strategies to Monitor and Engage Patients Prescribed Buprenorphine ^a^Significant difference between practice guidelines and prior Drug Addiction Treatment Act (DATA) waiver. ^b^Significant difference between practice guidelines and concurrent DATA waiver. ^c^Significant difference between prior DATA waiver and concurrent DATA waiver.

Among clinicians prescribing buprenorphine in the past 6 months, strategies to monitor and engage patients prescribed buprenorphine varied by waiver group (Figure 4). Practice guidelines clinicians compared with both prior and concurrent DATA waiver clinicians reported lower use of individual in-person counseling (220 [48.8%] vs 483 [66.3%; *P* < .001] and 206 [67.8%; *P* < .001]), group in-person counseling (33 [7.3%] vs 90 [12.3%; *P* = .002] and 44 [14.5%; *P* = .004]), individual remote telehealth counseling with (51 [11.3%] vs 199 [27.3%; *P* < .001] and 81 [26.6%; *P* < .001]) or without (41 [9.1%] vs 155 [21.3%; *P* < .001] and 54 [17.8%; *P* < .001]) video, in-person urine drug testing (152 [33.7%] vs 446 [61.2%; *P* < .001] and 191 [62.8%; *P* < .001]), in-person pill and film checks (45 [10.0%] vs 159 [21.8%; *P* < .001] and 71 [23.4%; *P* < .001]), and checking the state prescription drug monitoring program (PDMP) (226 [50.1%] vs 480 [65.8%; *P* < .001] and 188 [61.8%; *P* < .001]). Similar, but not significant, percentages of respondents reported prescribing naloxone for overdose prevention (224 [49.7%] vs 404 [55.4%; *P* = .06] and 150 [49.3%; *P* = .93]) and group remote telehealth counseling with (8 [1.8%] vs 26 [3.6%; *P* = .053] and 9 [3.0%; *P* = .30]) or without (2 [0.4%] vs 11 [1.5%; *P* = .053] and 6 [2.0%; *P* = .08]) video. Remote pill and film checks with video were reported less often among concurrent DATA waiver clinicians compared with prior DATA waiver clinicians (4 [1.3%] vs 26 [3.6%], respectively; *P* < .001).

## Discussion

To our knowledge, this survey study is the first to assess the similarities and differences of clinicians who obtained a DATA waiver under the education-exempted HHS buprenorphine practice guidelines released in April 2021 compared with clinicians who received a traditional DATA waiver prior to and after release of the practice guidelines. These findings are particularly salient given the recent federal legislative changes included in the fiscal year 2023 Congressional Omnibus Appropriation law that eliminates the requirement to obtain a DATA waiver in order to prescribe buprenorphine for OUD treatment.^13^ Practice guidelines clinicians were similarly exempted from DATA waiver educational requirements; therefore, the characteristics of these clinicians and their practice patterns may provide some insight into how the OUD treatment landscape is expected to evolve after DATA waiver elimination and may inform the need for complementary policy and education initiatives to expand access to MOUD.

Our findings suggest that the composition of clinicians opting into buprenorphine-based treatment may change as the regulatory requirements for prescribing buprenorphine evolve. For example, practice guidelines clinicians were more likely to be physicians, practice in ED or urgent care settings, and practice in urban areas. Our finding that more physicians as well as clinicians practicing in urban settings is consistent with prior research that examined trends in DATA-waivered clinicians during the COVID-19 pandemic and after release of the HHS practice guidelines in 2021.^12^ As the regulatory paradigm for buprenorphine prescribing evolves with the elimination of the DATA waiver, it will be important to track the types of clinicians prescribing buprenorphine, identify untapped pools of clinicians not engaging in prescribing, and tailor training and technical assistance accordingly.

Practice guidelines clinicians were less likely to incorporate other interventions and monitoring strategies in caring for patients with OUD compared with both traditional DATA waiver groups. Some of these differences were related to the larger percentage of ED and urgent care clinicians in the practice guidelines group (39.9%) compared with 17.0% and 12.5% in the prior and concurrent DATA waiver groups, respectively. In these settings, transient clinician-patient relationships make additional interventions, such as individual and group counseling, and monitoring strategies, including pill and film counts and conducting urine drug testing, less common compared with office-based settings. As restrictions on buprenorphine prescribing are further removed, it will be important to monitor practice patterns, quality of care, and treatment outcomes to assess intended and unintended outcomes of policy changes.

More immediately, regardless of waiver type, only approximately one-half of clinicians reported prescribing naloxone for overdose prevention to this high-risk population, a particularly concerning finding. The Centers for Disease Control and Prevention,^17^ SAMHSA,^18^ and US Food and Drug Administration^19^ recommend that clinicians should offer naloxone to patients receiving buprenorphine for OUD. In addition, slightly more than one-half of surveyed clinicians reported checking state PDMPs, which can help to identify high-risk concomitant medication use and other opportunities for clinical intervention. Educational and training programs, clinical practice guidelines, and other systems-level strategies should continue to prompt clinicians to prescribe naloxone and check PDMPs.

Our findings confirm that multiple barriers to wider adoption of buprenorphine treatment for OUD exist beyond removal of training requirements. Consistent with prior research,^3,5,9,10^ access to other services, including psychosocial services, and access to specialists, such as addiction medicine and psychiatric clinicians, remain as barriers to buprenorphine prescribing, even among clinicians who took the initial step to obtain a waiver. In addition, although practice guidelines clinicians responding to the survey reported less patient demand for buprenorphine than did traditionally waivered clinicians, all 3 groups noted lack of patient demand as the most common reason for not prescribing buprenorphine since obtaining a waiver. Furthermore, the fact that the majority of clinicians surveyed, regardless of waiver type, reported prescribing to 1 to 4 patients in an average month mirrors prior research showing that most DATA-waivered clinicians prescribe far below their patient limits.^20^ This underused treatment capacity may reflect challenges with patient engagement or interest, lack of screening for and identification of patients with OUD in clinical settings, and organizational and policy barriers that hinder clinicians’ efforts to incorporate OUD treatment into their practice.

Taken together, this study highlights the ongoing challenges with providing and engaging in buprenorphine treatment, even when an often-invoked barrier to prescribing, the educational requirement prior to obtaining a DATA waiver, is removed. Taking stock of these findings is paramount as the medical community and policy makers begin to implement actions in the context of DATA waiver elimination. It is hoped that these substantial policy changes will revolutionize the delivery of buprenorphine treatment. Our findings, coupled with recent research of waiver trends and buprenorphine prescribing after implementation of the education-exempted practice guidelines,^11^ suggest that removal of this barrier to entry for clinicians may not on its own necessarily translate to large-scale increases in buprenorphine prescribing against the backdrop of multiple clinician and patient challenges.

To fully optimize the outcomes of the DATA waiver elimination law, it will be critical to focus on clinician barriers, systems-level improvements, and payment policy changes that support models of care that integrate behavioral health services; incentivize MOUD; expand the substance use disorder workforce; counter clinician, patient, and institutional stigma; and provide training and technical assistance to support a broad range of clinicians in delivering care to patients with OUD and other substance use disorders. At the same time, policies, programs, and practices that holistically support patients to link to and engage in care are important.

This exploratory survey highlights several important topics for further investigation. First, the finding that clinicians practicing in ED or urgent care settings are overrepresented in the practice guidelines group suggests that removing training and administrative barriers to prescribing MOUD may be particularly important in this setting. Understanding the reasons for these differential increases and finding ways to enhance their positive association with patient outcomes following emergency medical care is a key next step. Second, understanding outcomes of patients treated under the practice guidelines is essential, as it may lend insight into how patient outcomes are expected to change after DATA waiver removal. Third, multiple surveys have found lack of patient demand as a key clinician-reported reason for not prescribing buprenorphine after obtaining a waiver.^9,10^ Future research should explore contributors to patient demand and patient-level drivers and disincentives of engagement in care.

### Limitations

This study has several limitations. First, although our response sample was large and incorporated variation with regard to demographic and clinical characteristics, the response rate was 19.5%, with 60.5% of respondents providing sufficient information to be included in this specific analysis; thus, findings may be subject to nonresponse bias. Second, the survey asked about a range of clinician barriers and strategies to treating patients; however, other barriers or strategies not assessed may exist. Third, while our survey instrument was largely drawn from prior surveys and expert review, some respondents might have misinterpreted some survey questions.

## Conclusions

In this survey study of clinicians recently receiving DATA waivers, we found that the education-exempted HHS practice guidelines brought in new clinicians, particularly physicians and clinicians practicing in ED and urgent care settings, although overall prescribing rates among this group were generally lower than among clinicians who obtained a traditional DATA waiver prior to or after release of the practice guidelines. Across all waiver approval types, we identified multiple barriers to buprenorphine prescribing and suboptimal use of strategies to manage risk among patients with OUD. As the medical community and policy makers begin to respond to legislation that has removed the DATA waiver, our findings suggest that it will be important that future efforts also focus on addressing the multiple challenges that constrain buprenorphine prescribing and engagement in care.

## Notes

Supplement 1. **eTable 1.** Survey Instrument **eFigure.** Flow Diagram of Final Study Population **eTable 2.** Geographic Distribution of Eligible Respondents and Actual Respondents by Waiver Approval Type

Supplement 2.
Data Sharing Statement
