# Supplementary material for: Characteristics and Prescribing Patterns of Clinicians Waivered to Prescribe Buprenorphine for Opioid Use Disorder Before and After Release of New Practice Guidelines
Source: JAMA Health Forum. 2023 Jul 21;4(7):e231982. doi: 10.1001/jamahealthforum.2023.1982 (PMC10362471; doi:10.1001/jamahealthforum.2023.1982)
Supplement: Supplement 1. — eTable 1. Survey Instrument eFigure. Flow Diagram of Final Study Population eTable 2. Geographic Distribution of Eligible Respondents and Actual Respondents by Waiver Approval Type [file jamahealthforum-e231982-s001.pdf]

Supplemental Online Content

Jones CM, Olsen Y, Ali MM, et al. Characteristics and prescribing patterns of clinicians waivered to prescribe buprenorphine for opioid use disorder before and after release of new practice guidelines. *JAMA Health Forum*. 2023;4(7):e231982. doi:10.1001/jamahealthforum.2023.1982

eTable 1. Survey Instrument

eFigure. Flow Diagram of Final Study Population

eTable 2. Geographic Distribution of Eligible Respondents and Actual Respondents by Waiver Approval Type

This supplemental material has been provided by the authors to give readers additional information about their work.

© 2023 Jones CM et al. *JAMA Health Forum.*

eTable 1. Survey Instrument

Provider/Practice Characteristics	Outcome	Code	Skip Logic	Notes
1. What is your gender identity?	Female	0	Go to 2	Select one
	Male	1		
	Nonbinary/Genderqueer/Gender Non-Conforming/Neither Exclusively Male Or Female	2		
	Other	3		
	Prefer not to respond to this question	-9		
2. What is your age group?	24 or younger	0	Go to 3	Select one
	25-34	1		
	35-44	2		
	45-54	3		
	55-64	4		
	65 or older	5		
	Prefer not to respond to this question	-9		
3. What is your race (please select all that apply)?	American Indian or Alaska Native	0	Go to 4	Select all that apply
	Asian	1		
	Black or African American	2		
	Native Hawaiian or Other Pacific Islander	3		
	White	4		
	Prefer not to respond to this question	-9		
4. What is your ethnicity?	Hispanic or Latino	0	Go to 5	Select one
	Not Hispanic or Latino	1		
	Prefer not to respond to this question	-9		
5. In what U.S. State or Territory is your primary practice location?	50 states, DC, PR, USVI, Guam, Other	0-55	Go to 6	Select one
	Prefer not to respond to this question	-9		
6. Is your PRIMARY practice location in an urban, suburban, or rural area?	Urban	0	Go to 7	Select one
	Suburban	1		
	Rural	2		
	Prefer not to respond to this question	-9		
7. What type of healthcare provider are you?	Physician (MD/DO)	0	Go to 8	Select one
	Nurse Practitioner or other nurse-eligible provider	1		
	Physician Assistant	2		
	Prefer not to respond to this question	-9		
8. What is your PRIMARY practice specialty? Select the one option that best describes your training and current practice specialty	Primary Care/Internal Medicine/Family Medicine	0	Go to 9	Select one
	Pediatrician	1		
	OB/GYN	2		
	Emergency Medicine	3		
	Psychiatry	4		
	Pain Medicine/Anesthesiology	5		
	Surgery	6		
	Neurology	7		

© 2023 Jones CM et al. *JAMA Health Forum.*

	Other (specify)	8		
	Prefer not to respond to this question	-9		
9. Do you have any of the following addiction medicine credentials?	Addiction Psychiatry (ABMS)	0	Go to 10	Select all that apply
	Addiction Medicine (ABAM/ABPM)	1		
	Addiction Medicine (AOA)	2		
	No, I do not have any of these addiction medicine credentials	3		
	Prefer not to respond to this question	-9		
10. Since completing your residency training (MD/DO) or qualifying training program (PA, NP or Other Nurse-eligible provider) how many years have you been in practice?	< 5 years	0	Go to 11	Select one
	5-10 years	1		
	11-15 years	2		
	16-19 years	3		
	20 years or more	4		
	Prefer not to respond to this question	-9		
11. What is your PRIMARY practice setting? Select the one practice setting where you spend more than 50% of your clinical practice time.	Office-based solo practice	0	Go to 12	Select one
	Office-based group practice	1		
	Specialty substance use treatment facility, not an Opioid Treatment Program	2		
	Opioid Treatment Program	3		
	Community clinic (FQHC, RHC, CCBHC)	4		
	Emergency Department	5		
	Urgent Care	6		
	Criminal Justice setting (incl. BOP)	7		
	In-patient/Hospital setting, Not Federal Government system	8		
	Veterans Health Administration (any type of facility)	9		
	Indian Health Service (any type of facility)	10		
	Department of Defense (any type of facility)	11		
	Other (specify)	12		
	Prefer not to respond to this question	-9		
12. What types of insurance do you accept in your practice?	Medicaid	0	Go to 13	Select all that apply
	Medicare	1		
	Private or Commercial Insurance	2		
	Workers Compensation	3		
	Other insurance	4		
	Do not accept any type of insurance in my practice	5		
	Prefer not to respond to this question	-9		
13. Have you given permission to be listed on SAMHSA's Buprenorphine Provider Locator?	No	0	Go to 14	Select one
	Yes	1		
	Do not know	2		
	Prefer not to respond to this question	-9		
14. Have you ever interacted with the	No	0	Go to 15	Select one
	Yes	1		

© 2023 Jones CM et al. *JAMA Health Forum.*

Providers Clinical Support System for Medication Assisted Treatment (PCSS-MAT)?	Do not know	2		
	Prefer not to respond to this question	-9		
15. Have you received education about buprenorphine for OUD treatment and/or OUD diagnosis and treatment from any of the following sources?	Medical/professional school teaching	0	Go to 16	Select all that apply
	Post graduate residency training	1		
	Fellowship training	2		
	Scientific conferences or continuing education courses	3		
	Self-directed reading of published literature or guidelines	4		
	Promotional materials or detailing from drug companies (not FDA REMS related materials)	5		
	FDA REMS materials related to buprenorphine products for OUD (e.g. REMS Instruction Letter to Prescribers, Medication Guide, Appropriate Use Checklist)	6		
	Colleagues (not as part of formal training or continuing education)	7		
	Other (specify)	8		
	None of the above	9		
	Prefer not to respond to this question	-9		
16. Traditionally, clinicians have only been able to prescribe buprenorphine for opioid use disorder (OUD) treatment after completing the educational training requirement and receiving a DATA 2000 waiver (referred to as the "Traditional DATA waiver process"). In April 2021, the US Department of Health and Human Services released the HHS Practice Guidelines for the Administration of Buprenorphine for Treatment of Opioid Use Disorder enabling clinicians to prescribe buprenorphine for OUD treatment for up	Traditional DATA waiver process	0	Go to Q17 if RESPONSE = 0<br>Go to Q33 if RESPONSE = 1<br>END OF SURVEY IF RESPONSE = 2 or -9	
	HHS Practice Guidelines process	1		
	Do not know	2		
	Prefer not to respond to this question	-9		

© 2023 Jones CM et al. *JAMA Health Forum.*

to 30 patients without completing the educational training requirement and obtaining the DATA 2000 waiver through the traditional process (referred to as the "HHS Practice Guidelines process")<br><br>Are you approved to prescribe buprenorphine under the traditional DATA waiver process or the HHS Practice Guidelines process?				
17. What is your highest DATA 2000 waiver patient limit currently?	30 patients	0	Go to 18	Select one
	100 patients	1		
	Prefer not to respond to this question	-9		
18. When did you obtain your initial DATA 2000 waiver?	In the 13 months between April 1, 2020 and April 28, 2021	0	Go to 19	Select one
	In the 7 months between May 1, 2021 and November 30, 2021	1		
	Not sure	2		
	Prefer not to respond to this question	-9		
19. Have you ever prescribed buprenorphine for OUD treatment since obtaining your DATA 2000 Waiver?	No	0	Go to Q20 IF RESPONSE = 0<br>Go to Q21 IF RESPONSE = 1<br>END SURVEY IF RESPONSE = -9	Select one
	Yes	1		
	Prefer not to respond to this question	-9		
20. What are the PRIMARY REASONS (Select Up to 3) you have not prescribed buprenorphine for OUD treatment since obtaining your DATA 2000 waiver (select up to three)?	Insufficient reimbursement from insurers	0	END OF SURVEY FOR RESPONDENTS ROUTED TO Q20	Select up to three
	Prior authorization and other insurance utilization management requirements	1		
	Lack of access to psychosocial services or other behavioral health providers for patients	2		
	Lack of access to addiction specialists for consultation	3		
	Lack of access to psychiatric services for patients with co-occurring mental health disorders	4		
	Concerns about managing patients with opioid use disorder	5		
	Lack of patient demand	6		

© 2023 Jones CM et al. *JAMA Health Forum.*

	Required to get DATA 2000 waiver for my job, but not interested in prescribing buprenorphine	7		
	Concerns about risks of misuse or diversion of buprenorphine	8		
	Prefer non-buprenorphine treatment options	9		
	Time constraints in my practice	10		
	Resistance from practice partners or staff or lack of institutional support	11		
	Concerns over DEA intrusion into my practice	12		
	Unable to find a supervising physician	13		
	Still setting up practice	14		
	None of the above	15		
	Prefer not to respond to this question	-9		
21. Have you prescribed buprenorphine for OUD treatment in the past 6 months?	No	0	Go to Q22 IF RESPONSE = 0 Go to Q23 IF RESPONSE = 1 END SURVEY IF RESPONSE = -9	Select one
	Yes	1		
	Prefer not to respond to this question	-9		
22. What are the PRIMARY REASONS (Select Up to 3) you have not prescribed buprenorphine for OUD treatment in the past 6 months?	Insufficient reimbursement from insurers	0	END OF SURVEY FOR RESPONDENTS ROUTEd TO Q22	Select up to three
	Prior authorization and other insurance utilization management requirements	1		
	Lack of access to psychosocial services or other behavioral health providers for patients	2		
	Lack of access to addiction specialists for consultation	3		
	Lack of access to psychiatric services for patients with co-occurring mental health disorders	4		
	Lack of confidence in managing patients with opioid use disorder	5		
	Lack of patient demand	6		
	No longer interested in prescribing buprenorphine for opioid use disorder treatment	7		
	Concerns about risks of misuse or diversion of buprenorphine	8		
	Prefer non-buprenorphine treatment options	9		
	Time constraints in my practice	10		
	Resistance from practice partners or staff or lack of institutional support	11		
	Concerns over DEA intrusion into my practice	12		
	Unable to find a supervising physician	13		
	Still setting up practice	14		
	None of the above	15		
	Prefer not to respond to this question	-9		
23. Think about the past 6 months. During this	0	0	Go to 24	Select one
	1-4	1		

© 2023 Jones CM et al. *JAMA Health Forum.*

time, how many patients have you prescribed buprenorphine for OUD treatment *in an average month*?	5-9	2		
	10-14	3		
	15-30	4		
	31-74	5		
	75-100	6		
	Prefer not to respond to this question	-9		
24. How many patients have you prescribed buprenorphine for OUD treatment to *in the past month?*	0	0	Go to 25	Select one
	1-4	1		
	5-9	2		
	10-14	3		
	15-30	4		
	31-74	5		
	75-100	6		
	Prefer not to respond to this question	-9		
25. Under the COVID-19 Public Health Emergency in March 2020, the Drug Enforcement Administration issued guidance allowing clinicians to prescribe buprenorphine to new patients without conducting an initial in-person examination.<br><br>Have you prescribed buprenorphine without conducting an in-person examination for a new patient (i.e., remote initiation) since DEA issued this guidance?	No	0	Go to Q26 IF RESPONSE = 0<br>Go to Q27 IF RESPONSE = 1<br>Go to Q31 IF RESPONSE = -9	Select one
	Yes	1		
	Prefer not to respond to this question	-9		
26. What barriers prevented you from prescribing buprenorphine without conducting an in-person examination?	At patient limit	0	Go to 31	Select all that apply
	No new patients	1		
	Inadequate broadband/internet in practice	2		
	Inadequate remote technology for patients	3		
	Prefer to see patients in person	4		
	Concern about home induction	5		
	Concern about buprenorphine diversion	6		
	Inadequate reimbursement for telemedicine services	7		
	Security or privacy concerns	8		
	Practice remains open for in-person visits	9		

© 2023 Jones CM et al. *JAMA Health Forum.*

	Limited information about remote induction protocols	10		
	None of the above	11		
	Prefer not to respond to this question	-9		
27. Did any new patients prescribed buprenorphine in the absence of an in-person examination (i.e., remote initiation) encounter problems related to buprenorphine induction?	No	0	Go to Q31 IF RESPONSE = 0,2 -9 Go to Q28 IF RESPONSE = 1	Select one
	Yes	1		
	Unknown	2		
	Prefer not to respond to this question	-9		
28. What induction-related problems did they encounter?	Significant withdrawal symptoms	0	Go to 29	Select all that apply
	Over-sedation	1		
	Allergic reaction	2		
	Other (specify)	3		
	Prefer not to respond to this question	-9		
29. Are the induction-related problems observed among patients initiating buprenorphine remotely in the absence of an in-person examination different than those experienced by patients who initiate in-person?	No	0	Go to Q31 IF RESPONSE = 0 or -9 Go to Q30 IF RESPONSE =1	Select one
	Yes	1		
	Prefer not to respond to this question	-9		
30. Please describe how the induction-related problems observed among patients initiating buprenorphine remotely were different than patients who initiate buprenorphine in-person	Open ended free text	Free text	Go to Q31	Free text
31. In the past 6 months, what strategies have you used to engage and monitor patients (select all that apply)?	Individual in-person counseling	0	Go to Q32	Select all that apply
	Group in-person counseling	1		
	Individual remote tele-health counseling without video	2		
	Individual remote tele-health counseling with video	3		

© 2023 Jones CM et al. *JAMA Health Forum.*

	Group remote tele-health counseling without video	4		
	Group remote tele-health counseling with video	5		
	In-person urine drug testing	6		
	In-person pill/film checks	7		
	Remote pill/film checks with video	8		
	Check state Prescription Drug Monitoring Program	9		
	Prescribe naloxone for overdose prevention	10		
	Other (specify)	11		
	None of the above	12		
	Prefer not to respond to this question	-9		
32. Which of the following are challenges that your patients have reported experiencing regarding opioid use disorder treatment in the past 6 months (select all that apply)?	No challenges reported	0	END OF SURVEY FOR RESPONDENTS ROUTED TO THIS QUESTION	Select all that apply
	Limited access to providers who are able to prescribe buprenorphine	1		
	Unreliable phone, computer, or internet service to engage with provider remotely	2		
	Inability to get medications for opioid use disorder treatment from pharmacy	3		
	Cost of treatment	4		
	In-person remote self-help or counseling groups have been cancelled	5		
	Unable to find remote self-help or counseling groups	6		
	Lack of transportation to doctor or pharmacy	7		
	Lack of access to psychosocial services or other behavioral health providers	8		
	Lack of access to addiction specialists for referral	9		
	Lack of access to psychiatric services for patients with co-occurring mental health disorders	10		
	Patient lacks stable housing	11		
	Other (specify)	12		
	None of the above	13		
	Prefer not to respond to this question	-9		
33. Prior to the HHS Practice Guidelines, what were the PRIMARY REASONS (Select Up to 3) you did not pursue obtaining a DATA 2000 waiver	Requirements for 8 hour training to obtain waiver for MDs/DOs and 24 hour training for PA/NP and other Nurse-eligible providers	0	ROUTED TO Q33 by RESPONSE = 1 to Q16<br>Go to Q34	Select up to three
	Insufficient reimbursement from insurers	1		
	Prior authorization and other insurance utilization management requirements	2		
	Lack of access to psychosocial services or other behavioral health providers for patients	3		
	Lack of access to addiction specialists for consultation	4		
	Lack of access to psychiatric services for patients with co-occurring mental health disorders	5		

© 2023 Jones CM et al. *JAMA Health Forum.*

	Concerns about managing patients with opioid use disorder	6		
	Lack of patient demand	7		
	Concerns about risks of misuse or diversion of buprenorphine	8		
	Prefer non-buprenorphine treatment options	9		
	Time constraints in my practice	10		
	Resistance from practice partners or staff or lack of institutional support	11		
	Concerns over DEA intrusion into my practice	12		
	Unable to find a supervising physician	13		
	Still setting up practice	14		
	None of the above	15		
	Prefer not to respond to this question	-9		
34. Have you prescribed buprenorphine for OUD treatment since your approval under the new HHS Practice Guidelines?	No	0	Go to Q35 IF RESPONSE = 0<br>Go to Q36 IF RESPONSE = 1<br>END SURVEY IF RESPONSE = -9	Select one
	Yes	1		
	Prefer not to respond to this question	-9		
35. What are the PRIMARY REASONS (Select Up to 3) you have not prescribed buprenorphine for OUD treatment since your approval under the new HHS Practice Guidelines?	Insufficient reimbursement from insurers	0	END OF SURVEY FOR RESPONDENTS ROUTED TO THIS QUESTION	Select up to three
	Prior authorization and other insurance utilization management requirements	1		
	Lack of access to psychosocial services or other behavioral health providers for patients	2		
	Lack of access to addiction specialists for consultation	3		
	Lack of access to psychiatric services for patients with co-occurring mental health disorders	4		
	Concerns about managing patients with opioid use disorder	5		
	Lack of patient demand	6		
	Required to get approved under the HHS Practice Guidelines for my job, but not interested in prescribing buprenorphine for OUD treatment	7		
	Concerns about risks of misuse or diversion of buprenorphine	8		
	Prefer non-buprenorphine treatment options	10		
	Time constraints in my practice	11		
	Resistance from practice partners or staff or lack of institutional support	12		
	Concerns over DEA intrusion into my practice	13		
	Unable to find a supervising physician	14		
	Still setting up practice	15		
	None of the above	16		
	Prefer not to respond to this question	-9		

© 2023 Jones CM et al. *JAMA Health Forum.*

36. Since you were approved under the HHS Practice Guidelines, how many patients have you prescribed buprenorphine for OUD treatment *in an average month?*	0	0	Go to Q37	Select one
	1-4	1		
	5-9	2		
	10-14	3		
	15-30	4		
	Prefer not to respond to this question	-9		
37. How many patients have you prescribed buprenorphine for OUD treatment to *in the past month?*	0	0	Go to Q38	Select one
	1-4	1		
	5-9	2		
	10-14	3		
	15-30	4		
	Prefer not to respond to this question	-9		
38. Under the COVID-19 Public Health Emergency in March 2020, the Drug Enforcement Administration issued guidance allowing clinicians to prescribe buprenorphine to new patients without conducting an initial in-person examination.<br><br>Have you prescribed buprenorphine without conducting an in-person examination for a new patient (i.e., remote initiation) since DEA issued this guidance?	No	0	Go to Q39 IF RESPONSE = 0<br>Go to Q40 IF RESPONSE = 1<br>Go to Q44 IF RESPONSE = -9	Select one
	Yes	1		
	Prefer not to respond to this question	-9		
39. What barriers prevented you from prescribing buprenorphine without conducting an in-person examination?	At patient limit	0	Go to Q44	Select all that apply
	No new patients	1		
	Inadequate broadband/internet in practice	2		
	Inadequate remote technology for patients	3		
	Prefer to see patients in person	4		
	Concern about home induction	5		
	Concern about buprenorphine diversion	6		
	Inadequate reimbursement for telemedicine services	7		
	Security or privacy concerns	8		
	Practice remains open for in-person visits	9		
	Limited information about remote induction protocols	10		
	None of the above	11		

© 2023 Jones CM et al. *JAMA Health Forum.*

	Prefer not to respond to this question	-9		
40. Did any new patients prescribed buprenorphine in the absence of an in-person examination (i.e., remote initiation) encounter problems related to buprenorphine induction?	No	0	Go to Q44 IF RESPONSE = 0, 2, -9 Go to Q41 IF RESPONSE = 1	Select one
	Yes	1		
	Unknown	2		
	Prefer not to respond to this question	-9		
41. What induction-related problems did they encounter?	Significant withdrawal symptoms	0	Go to Q42	Select all that apply
	Over-sedation	1		
	Allergic reaction	2		
	Other (specify)	3		
	Prefer not to respond to this question	-9		
42. Are the induction-related problems observed among patients initiating buprenorphine remotely in the absence of an in-person examination different than those experienced by patients who initiate in-person?	No	0	Go to Q44 IF RESPONSE = 0 or -9 Go to Q43 IF RESPONSE = 1	Select one
	Yes	1		
	Prefer not to answer this question	-9		
43. Please describe how the induction-related problems observed among patients initiating buprenorphine remotely were different than patients who initiate buprenorphine in-person.	Free text	Free Text	Go to Q44	Free Text
44. Since you were approved to prescribe buprenorphine for OUD treatment under the HHS Practice Guidelines, what strategies have you used to engage and monitor patients?	Individual in-person counseling	0	Go to Q45	Select all that apply
	Group in-person counseling	1		
	Individual remote tele-health counseling without video	2		
	Individual remote tele-health counseling with video	3		
	Group remote tele-health counseling without video	4		
	Group remote tele-health counseling with video	5		
	In-person urine drug testing	6		
	In-person pill/film checks	7		

© 2023 Jones CM et al. *JAMA Health Forum.*

	Remote pill/film checks with video	8		
	Check state Prescription Drug Monitoring Program	9		
	Prescribe naloxone for overdose prevention	10		
	Other (specify)	11		
	None of the above	12		
	Prefer not to respond to this question	-9		
45. Which of the following are challenges that your patients have reported experiencing regarding opioid use disorder treatment since you were approved under the HHS Practice Guidelines?	No challenges reported	0	END OF SURVEY FOR RESPONDENTS ROUTED TO THIS QUESTION	Select all that apply
	Limited access to providers who are able to prescribe buprenorphine	1		
	Unreliable phone, computer, or internet service to engage with provider remotely	2		
	Inability to get medications for opioid use disorder treatment from pharmacy	3		
	Cost of treatment	4		
	In-person remote self-help or counseling groups have been cancelled	5		
	Unable to find remote self-help or counseling groups	6		
	Lack of transportation to doctor or pharmacy	7		
	Lack of access to psychosocial services or other behavioral health providers	8		
	Lack of access to addiction specialists for referral	9		
	Lack of access to psychiatric services for patients with co-occurring mental health disorders	10		
	Patient lacks stable housing	11		
	Other (specify)	12		
	None of the above	13		
	Prefer not to respond to this question	-9		

© 2023 Jones CM et al. *JAMA Health Forum.*

eFigure. Flow Diagram of Final Study Population

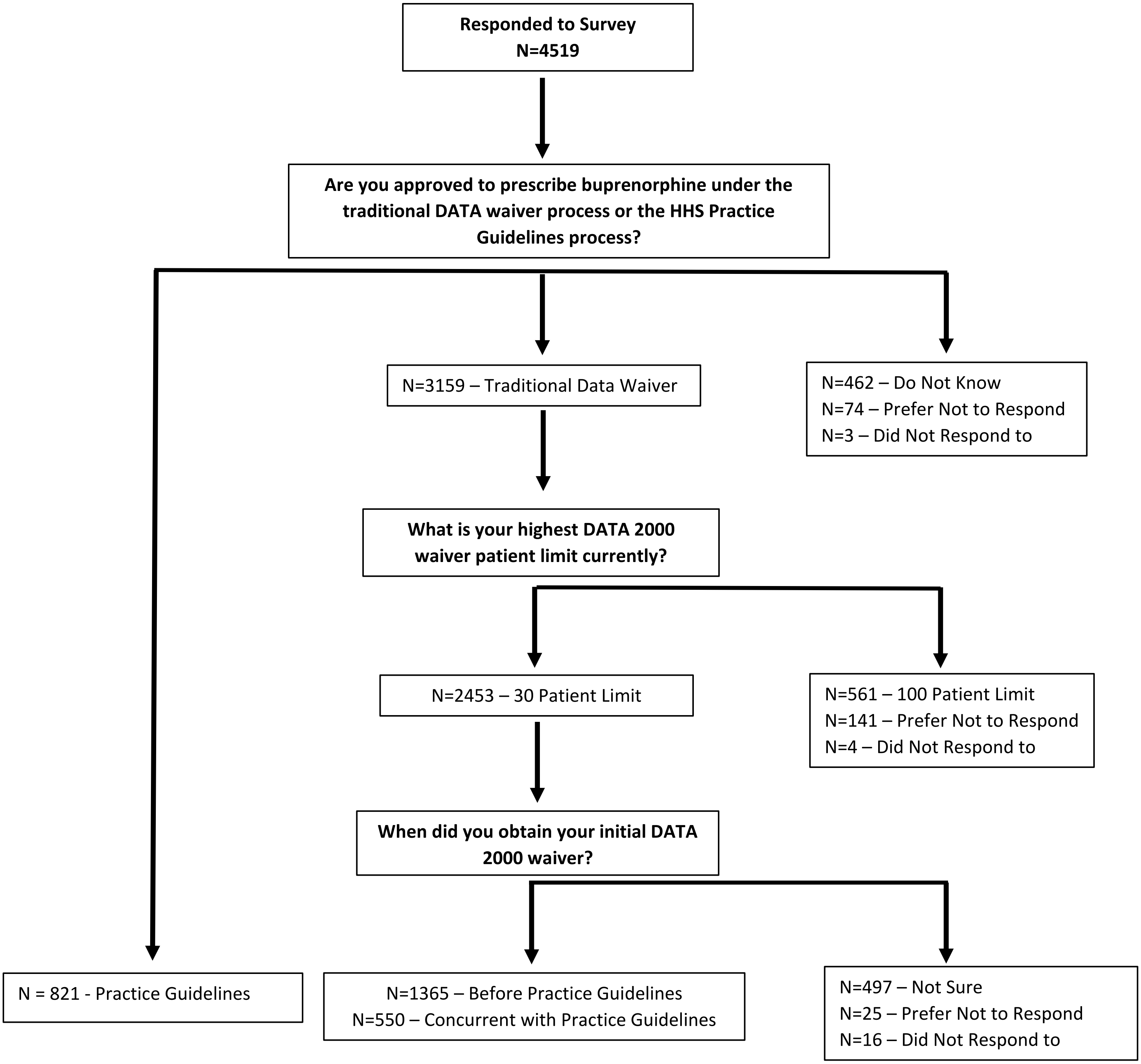

© 2023 Jones CM et al. *JAMA Health Forum.*

eTable 2. Geographic Distribution of Eligible Respondents and Actual Respondents by Waiver Approval Type.

State	Prior DATA Waiver		Concurrent DATA Waiver		Practice Guidelines		All DATA Waivers	
	% of Survey Sample	% of Eligible Clinicians	% of Survey Sample	% of Eligible Clinicians	% of Survey Sample	% of Eligible Clinicians	% of Survey Sample	% of Eligible Clinicians
AL	0.88	0.57	0.91	1.02	0.85	0.51	0.88	0.61
AK	0.78	0.74	0.55	0.52	0.97	0.79	0.79	0.73
AZ	3.01	2.03	1.83	2.53	2.44	0.92	2.67	1.79
AR	0.57	0.44	0.91	0.55	0.49	0.33	0.61	0.42
CA	11.80	13.77	11.33	12.24	19.73	23.62	13.70	16.28
CO	2.18	2.75	2.74	1.94	4.63	3.61	2.89	2.88
CT	1.35	1.39	1.10	1.39	0.85	0.84	1.18	1.24
DE	0.42	0.34	1.10	0.46	1.34	1.59	0.76	0.70
FL	3.85	2.88	3.84	3.98	2.19	2.72	3.43	2.98
GA	1.30	1.07	1.46	1.20	1.95	0.99	1.49	1.07
HI	0.26	0.38	1.10	0.40	0.12	0.19	0.36	0.33
ID	0.78	0.60	1.10	0.52	0.49	0.49	0.76	0.56
IL	3.27	4.34	5.12	3.67	2.19	2.76	3.31	3.82
IN	1.77	1.65	2.01	1.79	2.68	2.12	2.04	1.80
IA	0.57	0.48	0.55	0.55	0.12	0.39	0.46	0.47
KS	0.78	0.57	0.91	1.23	0.12	0.44	0.64	0.62
KY	1.98	1.32	0.91	1.57	0.85	0.90	1.52	1.24
LA	0.52	0.71	0.73	0.74	0.37	0.44	0.52	0.64
ME	1.04	0.89	0.73	1.05	0.49	0.77	0.85	0.88
MD	2.49	2.25	2.56	2.53	3.05	2.56	2.64	2.37
MA	2.34	3.70	4.02	4.01	3.05	4.06	2.79	3.84
MI	4.94	4.81	4.57	3.64	2.44	3.57	4.25	4.31
MN	3.27	2.65	3.84	2.71	2.44	1.98	3.16	2.47
MS	0.31	0.24	0.18	0.15	0.00	0.12	0.21	0.20

© 2023 Jones CM et al. *JAMA Health Forum.*

MO	1.92	1.12	1.46	1.48	1.95	1.75	1.85	1.34
MT	0.57	0.46	0.91	0.71	0.73	0.39	0.67	0.47
NE	0.52	0.57	0.37	0.34	0.24	0.10	0.43	0.41
NV	0.62	0.53	0.37	0.65	0.61	0.55	0.58	0.55
NH	0.94	0.99	0.73	0.65	0.73	0.81	0.85	0.90
NJ	2.39	2.43	2.56	2.71	2.44	2.49	2.43	2.48
NM	1.56	1.70	1.28	1.51	1.10	1.19	1.40	1.54
NY	5.77	6.75	5.30	6.69	4.99	5.50	5.50	6.40
NC	3.27	2.71	4.02	3.21	3.78	2.84	3.52	2.81
ND	0.26	0.24	0.73	0.18	0.61	0.16	0.43	0.21
OH	4.42	4.50	3.84	3.58	3.65	4.13	4.13	4.28
OK	0.68	0.75	1.28	0.77	0.61	0.35	0.76	0.64
OR	2.44	2.03	2.56	1.63	3.65	2.14	2.76	2.01
PA	4.63	5.50	4.02	4.65	4.51	4.83	4.50	5.20
RI	0.42	0.59	0.18	0.77	0.97	0.67	0.52	0.63
SC	1.66	1.37	1.46	1.57	1.46	1.11	1.58	1.32
SD	0.57	0.27	0.18	0.43	0.12	0.16	0.39	0.26
TN	1.61	1.48	1.46	1.14	0.73	0.71	1.37	1.22
TX	2.70	2.87	3.84	3.61	2.68	2.68	2.89	2.91
UT	1.51	1.05	1.28	1.08	0.73	0.96	1.28	1.03
VT	0.99	0.65	0.55	0.49	0.37	0.35	0.76	0.55
VA	2.23	1.96	1.83	2.00	2.68	2.20	2.28	2.03
WA	3.64	5.00	2.19	5.52	4.51	4.93	3.61	5.05
WV	0.62	0.67	0.73	0.43	0.00	0.17	0.49	0.50
WI	2.39	2.34	2.01	2.22	1.58	1.56	2.13	2.11
WY	0.26	0.18	0.73	0.28	0.12	0.17	0.30	0.19
DC	0.42	0.44	0.00	0.77	0.37	0.25	0.33	0.43
PR	0.36	0.24	0.00	0.43	0.24	0.09	0.27	0.22
Other	0.16	0.05	0.00	0.09	0.00	0.04	0.09	0.05
**Correlation**		**0.98**		**0.94**		**0.98**		**0.99**

© 2023 Jones CM et al. *JAMA Health Forum.*
