# Supplementary material for: Characteristics and Prescribing Patterns of Clinicians Waivered to Prescribe Buprenorphine for Opioid Use Disorder Before and After Release of New Practice Guidelines
Source: JAMA Health Forum. 2023 Jul 21;4(7):e231982. doi: 10.1001/jamahealthforum.2023.1982 (PMC10362471; doi:10.1001/jamahealthforum.2023.1982)
Supplement: Supplement 2. — Data Sharing Statement [file jamahealthforum-e231982-s002.pdf]

Data Sharing Statement

Jones. Characteristics and Prescribing Patterns of Clinicians Waivered to Prescribe Buprenorphine for Opioid Use Disorder Before and After Release of New Practice Guidelines. *JAMA Health Forum*. Published July 21, 2023. doi:10.1001/jamahealthforum.2023.1982

Data
Data available: No
